# Plasma biomarkers for amyloid, tau, and cytokines in Down syndrome and sporadic Alzheimer’s disease

**DOI:** 10.1186/s13195-019-0477-0

**Published:** 2019-03-21

**Authors:** Carla M. Startin, Nicholas J. Ashton, Sarah Hamburg, Rosalyn Hithersay, Frances K. Wiseman, Kin Y. Mok, John Hardy, Alberto Lleó, Simon Lovestone, Lucilla Parnetti, Henrik Zetterberg, Abdul Hye, LonDownS Consortium, Andre Strydom, Elizabeth Fisher, Dean Nizetic, John Hardy, Victor Tybulewicz, Annette Karmiloff-Smith, Tamara Al-Janabi, David Zhang, André Strydom

**Affiliations:** 1 Department of Forensic and Neurodevelopmental Sciences, Institute of Psychiatry, Psychology and Neuroscience, King’s College London, 16 De Crespigny Park, London, SE5 8AF UK; 2 Division of Psychiatry, University College London, London, UK; 3 The LonDownS Consortium (London Down Syndrome Consortium), London, UK; 4 Maurice Wohl Clinical Neuroscience, Institute of Psychiatry, Psychology and Neuroscience, King’s College London, London, UK; 5 NIHR Biomedical Research Centre for Mental Health, Biomedical Research Unit for Dementia at South London, and Maudsley NHS Foundation, London, UK; 6 Department of Psychiatry and Neurochemistry, Institute of Neuroscience and Physiology, Sahlgrenska Academy at the University of Gothenburg, Mölndal, Sweden; 7 Wallenberg Centre for Molecular & Translational Medicine, University of Gothenburg, Gothenburg, Sweden; 8 Department of Neurodegenerative Disease, Institute of Neurology, University College London, London, UK; 9 Department of Molecular Neuroscience, Institute of Neurology, University College London, London, UK; 10 Division of Life Science, Hong Kong University of Science and Technology, Hong Kong, SAR People’s Republic of China; 11 Reta Lila Weston Institute, Institute of Neurology, University College London, London, UK; 12 Memory Unit, Neurology Department, Hospital de la Santa Creu i Sant Pau, Barcelona, Spain; 13 Department of Psychiatry, University of Oxford, Oxford, UK; 14 Centre for Memory Disturbances, Laboratory of Clinical Neurochemistry, Section of Neurology, Department of Medicine, University of Perugia, Perugia, Italy; 15 Clinical Neurochemistry Laboratory, Sahlgrenska University Hospital, Mölndal, Sweden; 16 UK Dementia Research Institute at UCL, London, UK

**Keywords:** Down syndrome, Alzheimer’s disease, Dementia, Biomarker, Plasma, Amyloid, Tau, Interleukin 1β, Cytokines

## Abstract

**Background:**

Down syndrome (DS), caused by chromosome 21 trisomy, is associated with an ultra-high risk of dementia due to Alzheimer’s disease (AD), driven by amyloid precursor protein (*APP*) gene triplication. Understanding relevant molecular differences between those with DS, those with sporadic AD (sAD) without DS, and controls will aid in understanding AD development in DS. We explored group differences in plasma concentrations of amyloid-β peptides and tau (as their accumulation is a characteristic feature of AD) and cytokines (as the inflammatory response has been implicated in AD development, and immune dysfunction is common in DS).

**Methods:**

We used ultrasensitive assays to compare plasma concentrations of the amyloid-β peptides Aβ_40_ and Aβ_42_, total tau (t-tau), and the cytokines IL1β, IL10, IL6, and TNFα between adults with DS (*n* = 31), adults with sAD (*n* = 27), and controls age-matched to the group with DS (*n* = 27), and explored relationships between molecular concentrations and with age within each group. In the group with DS, we also explored relationships with neurofilament light (NfL) concentration, due to its potential use as a biomarker for AD in DS.

**Results:**

Aβ_40_, Aβ_42_, and IL1β concentrations were higher in DS, with a higher Aβ_42_/Aβ_40_ ratio in controls. The group with DS showed moderate positive associations between concentrations of t-tau and both Aβ_42_ and IL1β. Only NfL concentration in the group with DS showed a significant positive association with age.

**Conclusions:**

Concentrations of Aβ_40_ and Aβ_42_ were much higher in adults with DS than in other groups, reflecting *APP* gene triplication, while no difference in the Aβ_42_/Aβ_40_ ratio between those with DS and sAD may indicate similar processing and deposition of Aβ_40_ and Aβ_42_ in these groups. Higher concentrations of IL1β in DS may reflect an increased vulnerability to infections and/or an increased prevalence of autoimmune disorders, while the positive association between IL1β and t-tau in DS may indicate IL1β is associated with neurodegeneration. Finally, NfL concentration may be the most suitable biomarker for dementia progression in DS. The identification of such a biomarker is important to improve the detection of dementia and monitor its progression, and for designing clinical intervention studies.

## Background

Down syndrome (DS) is associated with an ultra-high risk of developing dementia caused by Alzheimer’s disease (AD) [1]. The lifetime dementia risk in DS is in excess of 95%, with a median age of onset of 55 years [2, 3], and the majority of adults with DS who die after the age of 36 have a diagnosis of dementia [4]. DS is caused by chromosome 21 trisomy and is the most common genetic cause of intellectual disability, with a UK incidence of approximately 1 in 1000 live births [5]. AD in DS is driven by the overexpression of genes on chromosome 21 due to their triplication, with triplication of the amyloid precursor protein (*APP*) gene being of particular importance. The amyloid precursor protein encoded by the *APP* gene is cleaved by β and γ secretases to form amyloid-β (Aβ) peptides, with Aβ_42_ the main component of the Aβ plaques characteristic of AD [6]. Such Aβ deposits are found in the brains of adults with full trisomy 21 by the mid-30s [1, 7]. Further, familial mutations in, or duplications of, the *APP* gene cause early-onset AD independent of DS [1, 8], and individuals with a partial trisomy of chromosome 21 not including *APP* do not develop AD or show Aβ pathology [9, 10]. Triplication of the *APP* gene and the subsequent overproduction of Aβ peptides are therefore likely to be central to the development of AD in people with DS, and DS may be considered a genetic cause of AD.

The presence of Aβ plaques in both sporadic AD (sAD) without DS and in people with DS, with the high risk of dementia in DS, indicates their development is a key component of the development of AD. Indeed, the amyloid hypothesis of AD states the presence of Aβ plaques initiates a cascade of events that leads to cell death and cognitive decline [11, 12]. Other pathophysiological mechanisms are also important within this cascade.

Firstly, the development of neurofibrillary tangles, composed of hyperphosphorylated and misfolded tau protein, is another neuropathological feature of AD. There is a stronger association between cognitive decline and tau pathology than with Aβ plaques in both DS [13] and sAD [14]. Secondly, neuronal damage within the brain can be measured by the release of neurofilament light (NfL), a scaffolding cytoskeleton protein, into plasma in a number of neurodegenerative disorders including sAD [15] and AD in DS [16, 17]. Finally, the inflammatory response is of interest as a potential contributory molecular mechanism to the development of AD. In sAD, the role of the immune system is supported by epidemiological and genetic studies. The presence of systemic infections and increased blood (plasma or serum) concentrations of cytokines including interleukin 1β (IL1β), interleukin 10 (IL10), interleukin 6 (IL6), and tumour necrosis factor α (TNFα) have been associated with sAD and predict cognitive decline [18–22]. Genome-wide association studies of sAD have implicated several genes involved in immune function (including variants in *CLU*, *ABCA7*, *CR1*, and *CD33*) [23–25], and there is an over-representation of genetic associations with sAD in pathways involved in the innate immune response [26].

To fully understand the development of AD in DS, it is therefore important to understand relevant molecular differences between those with DS, those with sAD, and controls. Similarities and group differences in plasma concentrations of molecules associated with AD and relationships between these possible biomarkers, in particular Aβ peptides and tau, may help to identify markers of AD progression. Previous studies comparing plasma concentrations of Aβ peptides and tau in individuals with DS to age-matched controls have reported increased concentrations of Aβ_40_ and Aβ_42_ [17, 27–37] and total tau (t-tau) [28, 38] in DS. Results regarding the Aβ_42_/Aβ_40_ ratio are less consistent; while several studies have reported this to be lower in those with DS [28, 30, 31], others have reported this to be higher [33] or to show no difference [29] relative to controls. To date, few studies have compared plasma concentrations of Aβ peptides and tau in adults with DS to those with sAD. Two studies have reported increased Aβ_40_ and Aβ_42_ in those with DS [29, 36], while another study reported lower concentrations of both Aβ_42_ and t-tau in adults with DS [28] compared to individuals with sAD.

In addition, a potential involvement of the immune system in the development of AD is of particular interest in DS due to immune dysfunction being common in DS, with an increased vulnerability to some types of infections throughout life [39] and higher rates of autoimmune disorders [40] in DS. Further, in DS, there is an overexpression of immune genes found on chromosome 21 (such as *IFNAR1*, *IFNAR2*, and *IFNGR2*, which all encode interferon-γ (IFN-γ) receptor proteins) [41]. In adults and children with DS, higher blood (plasma or serum) concentrations of IL10 [31, 42], IL6 [31, 43, 44], and TNFα [31, 43, 45] have been reported compared to age-matched controls, although lower concentrations of IL10 [45], IL6 [42], and TNFα [42] in DS have also been reported. A recent meta-analysis of 19 cytokine studies in adults and children with DS suggested that IL1β, TNFα, and IFN-γ (but not IL6 or IL10) concentrations are raised by trisomy 21 [46].

Further studies using newly developed ultrasensitive assays are therefore needed to better understand differences in concentrations of Aβ peptides, tau, and cytokines in individuals with DS, sAD, and controls. We therefore compared plasma concentrations of Aβ_40_, Aβ_42_, t-tau, IL1β, IL10, IL6, and TNFα between adults with DS, adults with sAD, and controls age-matched to the group with DS (to determine the effect of triplication of chromosome 21), and explored relationships between molecular concentrations within each group. Given the universal development of AD pathology in those with DS and increased risk of dementia with increased age [2], we also explored relationships between molecular concentrations and age. In addition, in the group with DS, we explored relationships with concentrations of NfL. Our group and others have previously shown NfL to be a potential biomarker for AD in DS, with higher NfL concentration associated with increased age and dementia development [16, 17], and so molecular concentrations that correlate with NfL may be particularly informative.

## Methods

### Participants

Adults in the group with DS (*n* = 31, including 7 with a clinical diagnosis of dementia) were recruited as a part of the LonDownS Consortium’s studies in adults with DS [47]. Clinical diagnoses of dementia were made by each individual’s clinician after a comprehensive clinical assessment. Participants were included in the present study if they lived within travelling distance of London to allow processing time for the blood sample, and they agreed to have a blood test. The presence of an additional chromosome 21 was confirmed genetically using saliva or blood samples; following DNA extraction, genome-wide single nucleotide polymorphism genotyping was performed using an Illumina OmniExpressExome array (San Diego, CA, USA) at UCL Genomics, then assembled and visually inspected in GenomeStudio. All adults were observed to have trisomy 21, with clinical notes from two individuals confirming this was due to a translocation.

For adults with DS, ethical approval was obtained from the North West Wales Research Ethics Committee (13/WA/0194). Where individuals had the capacity to consent for themselves, we obtained written informed consent. Where individuals did not have the capacity to consent, a consultee was asked to approve the individual’s inclusion based on their knowledge of the individual and his/her wishes, in accordance with the UK Mental Capacity Act 2005.

Adults in the sAD and control groups (*n* = 27 per group) were recruited from the multi-centre consortium European Medical Informatics Framework (EMIF) [48]. All plasma samples used in this study were from two sites (Clinica Neurologica, Universita di Perugia, and Hospital de la Santa Creu i Sant Pau, Barcelona). All participants in the sAD group had an AD diagnosis according to standard criteria [49], and biological AD was also confirmed using concentrations of cerebrospinal fluid (CSF) Aβ_42_ with a cutoff value indicating AD (< 800 pg/ml and < 550 pg/ml for Perugia and Barcelona, respectively) [50, 51]. All individuals in the sAD and control groups had Mini-Mental State Examination (MMSE) scores available. Where possible participants in the sAD and control groups were matched in age and gender to participants with DS; age matching was not fully possible for the group with sAD due to the older age of this group (see Table 1).

**Table 1:** Group demographics

	DS	sAD	Controls	Group comparison
*n*	31	27	27	N/A
Age (years)	46.77 ± 10.99 (23–67)	59.33 ± 4.04 (51–67)	49.26 ± 10.40 (24–64)	*F*(2,82) = 14.84, *p* < 0.001^a^
Sex	9 (29.0%) females, 22 (71.0%) males	9 (33.3%) females, 18 (66.7%) males	11 (40.7%) females, 16 (59.3%) males	*Χ*(2) = 0.89, *p* = 0.641
Intellectual disability severity^b^	17 (54.8%) mild, 11 (35.5%) moderate, 3 (9.7%) severe	N/A	N/A	N/A
*APOE* ε4 carrier	8 (25.8%) carriers, 23 (74.2%) non-carriers	10 (37.0%) carriers, 17 (63.0%) non-carriers	5 (18.5%) carriers, 22 (81.5%) non-carriers	*Χ*(2) = 2.38, *p* = 0.304
Mini-Mental State Examination (MMSE) score	N/A	19.19 ± 4.42 (7–26)	28.89 ± 1.12 (27–30)	*t*(52) = −11.06, *p* < 0.001^c^

Values for age and MMSE score given as mean ± standard deviation (range)

^a^sAD group older than DS and control groups (both *p* < 0.001), no difference between DS and control groups (*p* = 0.915)

^b^Pre-dementia level, assessed via informant report based on everyday functional descriptions

^c^sAD compared to controls only

For adults with sAD and controls, ethical approval was obtained from the regional ethics committee. Plasma and CSF samples were routinely collected in all subjects undergoing a diagnostic work-up for suspected neurodegenerative diseases, with informed written consent obtained for their use in research from patients or their representatives.

### APOE genotyping

*APOE* genotype was determined using a Thermo Fisher Scientific TaqMan assay for SNPs rs7412 and rs429358 (Waltham, MA, USA).

### Sample processing and assays

Blood samples from individuals with DS were collected in EDTA tubes after participants had undergone a cognitive assessment [47] and processed as quickly as feasible (within approximately 3 h). Plasma was prepared by centrifuging samples for 10 min at 2200 g, then the supernatant was aliquoted and stored at − 80 °C. Samples were only thawed immediately prior to analysis. Due to difficulties in obtaining blood samples from this population, we had to be pragmatic about the practicalities of collecting these samples. Blood samples were collected at a range of times throughout the day, with samples collected between 9.30 am and 5.40 pm and the majority of samples collected after midday (mean time of collection = 2.33 pm, standard deviation = 1.99 h). It was not possible to take fasting blood samples for research purposes, though blood samples were typically taken at the end of a cognitive assessment that had lasted several hours, and participants did not eat during this time. In addition, the amount of time samples were in the freezer before analysis varied as it took a number of months to collect enough blood samples for our analysis. During this time, samples remained frozen at − 80 °C. To assess potential relationships between the number of months samples were frozen before analysis and the concentrations of each molecule, we performed correlational analyses; there were no significant relationships observed (results not shown).

Blood sample collection and processing methods for adults in the sAD and control groups from the EMIF cohort have been previously described [48].

For all three groups, plasma concentrations of Aβ_40_, Aβ_42_, and t-tau (Human Neurology 3-Plex A assay (N3PA)), IL1β (Human IL-1β 2.0), and IL10, IL6, and TNFα (Human Cytokine 3-Plex A) were measured in duplicate using ultrasensitive Simoa immunoassays (Quanterix, Lexington, MA, USA) according to the manufacturer’s instructions at the Institute of Psychiatry, Psychology and Neuroscience, King’s College London. Samples from the three groups were blinded and randomised across two analytical plates for each assay. All duplicate measures for all targets had an average coefficient of variation (CV) of between 4 and 8%.

For the group with DS, concentrations of NfL were previously obtained from samples taken at the same time [16]. Blood samples collected in lithium heparin tubes were first layered over a similar amount of Ficoll (GE Healthcare, Little Chalfont, UK), centrifuged for 40 min at 400 g, then the supernatant was aliquoted and stored at − 80 °C until analysis. Plasma NfL concentration was measured using the Simoa immunoassay (Human NF-light) (Quanterix, Lexington, MA, USA) according to the manufacturer’s instructions at the Institute of Neurology, University College London. Values used in the present study had a median value of 27.09 pg/ml (range 10.97–112.60, *n* = 26).

### Statistical analysis

SPSS version 22 was used for analyses. Age and demographic factors were compared between groups using ANOVAs, two-sample *t* tests, or chi-squared tests as appropriate. To compare concentrations of molecules between groups of adults with DS, sAD, and controls, we performed ANCOVAs for log-transformed concentrations (due to concentrations not being normally distributed) with age, sex, and the presence of an *APOE* ε4 allele included as covariates to adjust for their potentially confounding effects, with *η*^2^ values determining the overall effect size of group and post hoc pairwise comparisons with Bonferroni corrections applied where appropriate. Due to concentrations not being normally distributed, non-parametric Spearman’s rank correlational analysis was performed for each group separately to determine associations between concentrations of molecules and with age, in addition to associations with NfL concentration for the group with DS. Due to the number of correlations performed, correlation coefficients were used to determine relationships rather than *p* values, with only strong and moderate correlations considered; these were defined as absolute values of correlation coefficients of 0.70 and above, and between 0.50 and 0.69, respectively.

## Results

**Fig. 1:**
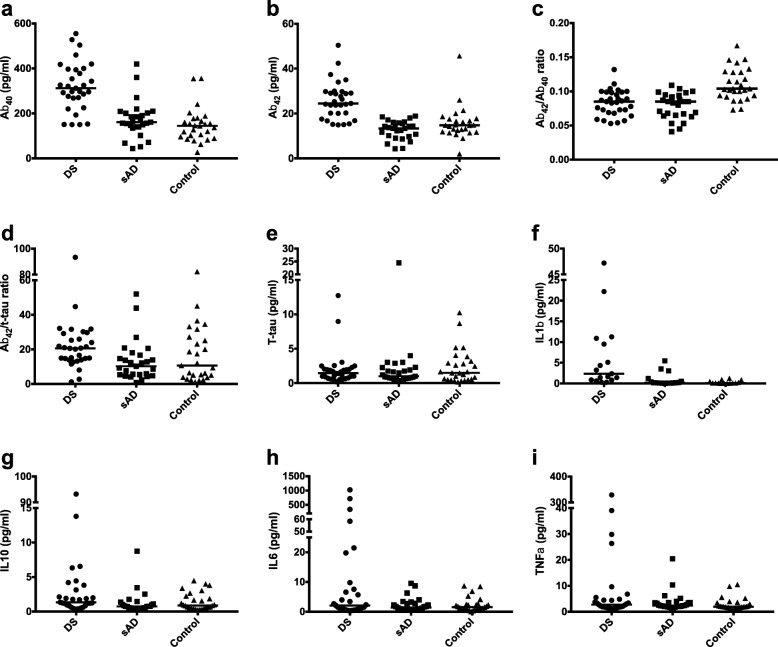
Concentrations of biomarkers for each group. **a** Aβ_40_, **b** Aβ_42_, **c** Aβ_42_/Aβ_40_, **d** Aβ_42_/t-tau, **e** t-tau, **f** IL1β, **g** IL10, **h** IL6, and **i** TNFα. Lines indicate median value (pg/ml)

**Fig. 2:**
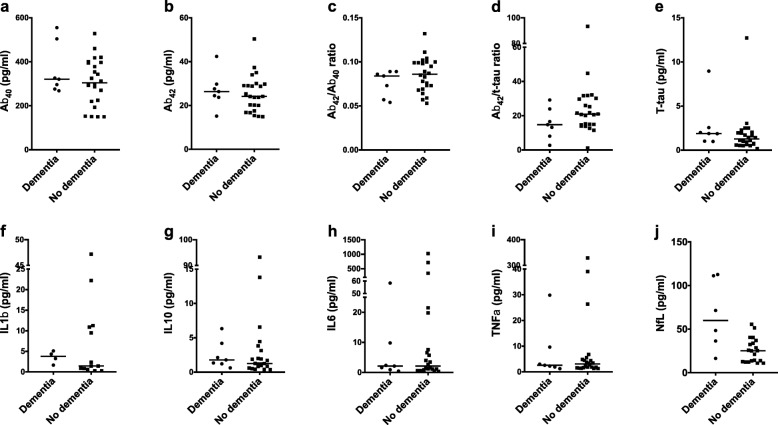
Concentrations of biomarkers for adults with DS with and without clinical dementia. **a** Aβ_40_, **b** Aβ_42_, **c** Aβ_42_/Aβ_40_, **d** Aβ_42_/t-tau, **e** t-tau, **f** IL1β, **g** IL10, **h** IL6, **i** TNFα, and **j** NfL. Lines indicate median value (pg/ml)

Demographic information for individuals in the three groups can be seen in Table 1, with median values and ranges for molecule concentrations in Table 2 and Fig. 1. As expected, the group with sAD was significantly older than the group with DS and the control group. One plate for IL1β failed (containing 14 DS, 11 sAD, and 13 control samples), resulting in fewer samples measured for IL1β. Demographic information and median values and ranges for molecule concentrations for adults with DS split into subgroups with and without clinical dementia can be found in Table 3 and Fig. 2. Statistical analysis was not performed to compare these subgroups due to small sample sizes.

**Table 2:** Concentrations of biomarkers for each group and group comparisons

	DS	sAD	Controls	ANCOVA	Post hoc
Aβ_40_	312.00 (150.24–555.00)	160.80 (43.60–420.00)	144.40 (26.88–355.60)	*F*(2,79) = 24.28, *p* < 0.001, *η*^2^ = 0.38	DS vs sAD *p* < 0.001 DS vs controls *p* < 0.001 sAD vs controls *p* = 0.506
Aβ_42_	24.48 (14.92–50.40)	13.32 (4.28–18.84)	14.76 (2.00–45.62)	*F*(2,79) = 20.36, *p* < 0.001, *η*^2^ = 0.34	DS vs sAD *p* < 0.001 DS vs controls *p* < 0.001 sAD vs controls *p* = 0.710
Aβ_42_/Aβ_40_	0.09 (0.05–0.13)	0.08 (0.04–0.11)	0.10 (0.07–0.17)	*F*(2,79) = 15.43, *p* < 0.001, *η*^2^ = 0.28	DS vs sAD *p* = 1.000 DS vs controls *p* < 0.001 sAD vs controls *p* < 0.001
Aβ_42_/t-tau	20.60 (1.17–93.33)	10.23 (0.77–52.00)	10.59 (1.14–82.25)	*F*(2,79) = 2.30, *p* = 0.107, *η*^2^ = 0.06	N/A
T-tau	1.45 (0.18–12.72)	1.00 (0.33–24.48)	1.49 (0.16–10.24)	*F*(2,79) = 0.64, *p* = 0.529, *η*^2^ = 0.02	N/A
IL1β^a^	2.35 (0.27–47.20)	0.18 (0.02–5.45)	0.09 (0.00–1.25)	*F*(2,41) = 13.84, *p* < 0.001, *η*^2^ = 0.40	DS vs sAD *p* = 0.002 DS vs controls *p* < 0.001 sAD vs controls *p* = 1.000
IL10^b^	1.34 (0.36–93.20)	0.75 (0.27–8.76)	0.87 (0.27–4.48)	*F*(2,76) = 2.38, *p* = 0.100, *η*^2^ = 0.06	N/A
IL6^b^	2.12 (0.36–1024.00)	1.45 (0.50–9.56)	1.54 (0.07–8.70)	*F*(2,76) = 2.54, *p* = 0.086, *η*^2^ = 0.06	N/A
TNFα^b^	2.76 (1.25–328.80)	2.37 (1.36–20.44)	1.97 (0.56–10.50)	*F*(2,76) = 2.42, *p* = 0.096, η^2^ = 0.06	N/A

Concentrations (pg/ml) given are median (range). ANCOVA performed on log-transformed values and including age, sex, and the presence of an *APOE* ε4 allele as covariates

^a^One plate failed (14 DS, 11 sAD, 13 controls)

^b^Three samples failed (2 DS and 1 sAD)

**Table 3:** Demographic information and concentrations of biomarkers for adults with DS with and without clinical dementia

	Dementia	No dementia
*n*	7	24
Age (years)	52.00 ± 10.36 (40–67)	45.25 ± 10.90 (23–65)
Sex	2 (28.6%) females, 5 (71.4%) males	7 (29.2%) females, 17 (70.8%) males
Intellectual disability severity^a^	5 (71.4%) mild, 1 (14.3%) moderate, 1 (14.3%) severe	12 (50.0%) mild, 10 (41.7%) moderate, 2 (8.3%) severe
*APOE* ε4 carrier	4 (57.1%) carriers, 3 (42.9%) non-carriers	4 (16.7%) carriers, 20 (83.3%) non-carriers
Aβ_40_	320.80 (268.20–555.00)	304.80 (150.24–528.00)
Aβ_42_	26.36 (15.16–42.40)	24.14 (14.92–50.40)
Aβ_42_/Aβ_40_	0.08 (0.05–0.09)	0.09 (0.05–0.13)
Aβ_42_/t-tau	14.76 (2.73–29.18)	20.86 (1.17–93.33)
T-tau	1.88 (0.99–8.96)	1.27 (0.18–12.72)
IL1β^b^	3.79 (1.65–5.10)	1.44 (0.27–47.20)
IL10^c^	1.78 (0.62–6.33)	1.26 (0.36–93.20)
IL6^c^	2.12 (0.44–58.40)	2.12 (0.36–1024.00)
TNFα^c^	2.58 (1.25–29.84)	3.08 (1.30–328.80)
NfL^d^	59.84 (16.36–112.60)	25.10 (10.97–55.45)

Values for age given as mean ± standard deviation (range), and concentrations (pg/ml) given are median (range)

^a^Pre-dementia level, assessed via informant report based on everyday functional descriptions

^b^One plate failed (3 with dementia, 11 without dementia)

^c^Two samples failed (both without dementia)

^d^Five samples missing (1 with dementia, 4 without dementia)

Concentrations of Aβ_40_, Aβ_42_, and IL1β were higher for individuals with DS compared to both those with sAD and controls, with median concentrations of Aβ_40_ and Aβ_42_ increased approximately two-fold and median concentration of IL1β increased over ten-fold in the group with DS. The Aβ_42_/Aβ_40_ ratio was higher for controls compared to both individuals with DS and those with sAD (Table 2).

**Fig. 3:**
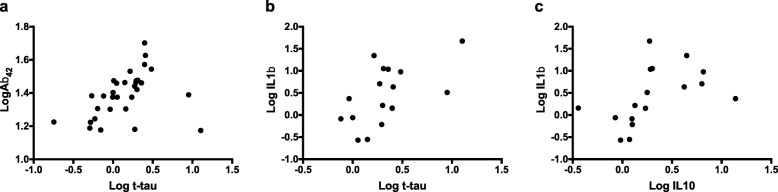
Relationships between biomarkers for adults with DS. **a** Log Aβ_42_ and log t-tau, **b** log t-tau and log IL1β, and **c** log IL1β and log IL10

All three groups showed strong positive associations between Aβ_40_ and Aβ_42_ concentrations (see Table 4). Both the groups with DS and with sAD showed a moderate positive association between IL10 and TNFα concentrations. In addition, the control group showed a moderate negative association between the Aβ_42_/Aβ_40_ ratio and IL10 concentration. Adults with DS showed several additional associations between concentrations of molecules (Table 4 and Fig. 3). Firstly, within this group, there was a moderate positive association between Aβ_42_ and t-tau concentrations. Secondly, within the cytokines investigated, there was a strong positive association between IL1β and IL10, and a moderate positive association between IL6 and TNFα. Finally, IL1β concentration showed a moderate positive association with t-tau concentration and a moderate negative association with the Aβ_42_/t-tau ratio. In addition, in the group with DS, only NfL concentration showed a moderate positive association with age, as previously published [16]. There were no other moderate or strong associations for molecular concentrations or with age across any group.

**Table 4:**
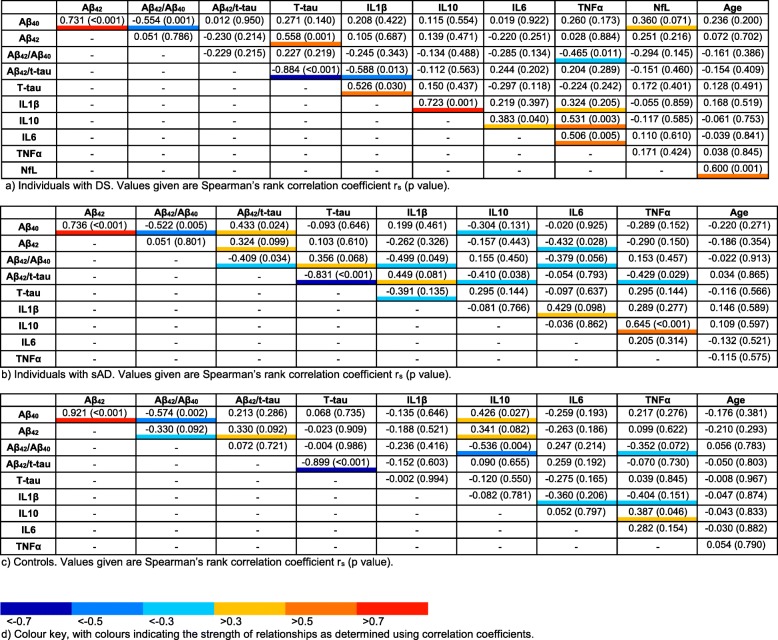
Relationships between concentrations of biomarkers for each group

## Discussion

We used ultrasensitive assays to compare plasma concentrations of Aβ peptides, tau, and selected cytokines between adults with DS, adults with sAD, and controls who were age-matched to the group with DS. We found significantly higher concentrations of Aβ_40_, Aβ_42_, and IL1β in those with DS compared to those with sAD and controls. The Aβ_42_/Aβ_40_ ratio was higher in controls compared to those with DS or sAD, indicating higher plasma concentrations of Aβ_42_ relative to concentrations of Aβ_40_ in controls. These group differences were observed when we accounted for age, sex, and the presence of an *APOE* ε4 allele.

Several positive associations were found between concentrations of different cytokines, in particular for the group with DS, possibly reflecting a more activated immune system in DS which may be associated with higher rates of infection and/or autoimmune disorders [39, 40]. The group with DS also showed positive associations between concentrations of t-tau and both Aβ_42_ and IL1β, and a negative association between IL1β and the Aβ_42_/t-tau ratio, while the control group showed a negative association between concentration of IL10 and the Aβ_42_/Aβ_40_ ratio.

Results relating to concentrations of Aβ peptides and their ratio indicate both likely differences and similarities in the development of AD between those with DS and those with sAD. Similar to the results of previous studies, we observed higher concentrations of Aβ_40_ and Aβ_42_ in those with DS compared to those with sAD and controls [17, 27–37]. This observation is likely due to triplication of the *APP* gene in adults with DS leading to elevated production of Aβ peptides. Concentrations of Aβ_40_ and Aβ_42_ in adults with DS were approximately double those in the other groups, rather than 1.5 times higher as may be expected based on the presence of an additional *APP* gene alone, suggesting that factor(s) other than *APP* triplication may contribute to increased Aβ concentrations [52], such as a positive feedback loop or an overloaded perivascular drainage system. Although concentrations of Aβ_40_ and Aβ_42_ were higher in those with DS compared to those with sAD, there was no difference in the Aβ_42_/Aβ_40_ ratio between these two groups. This may suggest the relative processing and deposition of Aβ_40_ and Aβ_42_ in the brain is similar between the two groups. The higher concentrations of Aβ_40_ and Aβ_42_ in adults with DS as compared to those with sAD, and no difference in the Aβ_42_/Aβ_40_ ratio between the two groups, is despite the younger age of the group with DS compared to those with sAD. In contrast, the Aβ_42_/Aβ_40_ ratio was higher in controls, indicating this group has a higher relative plasma Aβ_42_ concentration and/or a lower relative plasma Aβ_40_ concentration compared to the other two groups. This may be due to the build-up of Aβ plaques in the brains of those with DS and those with sAD, with Aβ_42_ a main component, resulting in a relative decrease of free Aβ_42_ and a lower Aβ_42_/Aβ_40_ ratio. Similarly, previous studies have associated a lower Aβ_42_/Aβ_40_ ratio with a higher risk of developing dementia [53] and with increased Aβ accumulation in the brain as measured using Aβ positron emission tomography [54–57].

Adults with DS also showed significantly higher concentrations of IL1β compared to adults with sAD and controls [46], and in the group with DS, IL1β concentration was positively correlated with t-tau concentration and negatively correlated with the Aβ_42_/t-tau ratio. Given the lack of an association between concentrations of IL1β and Aβ_42_ in this group, it is likely the latter association is driven by the association with t-tau. IL1β is a pro-inflammatory cytokine produced by activated macrophages, and previous studies have indicated increased concentrations of IL1β are associated with AD [20] and precede cognitive decline [19]. The increase in IL1β concentration in DS may reflect increased vulnerability to peripheral infections [39] and/or increased prevalence of autoimmune disorders in those with DS [40]. Plasma t-tau concentration is associated with severity of neurodegeneration, thought to be due to neuronal damage causing tau release from the brain [58]. The association between IL1β and plasma t-tau in DS may therefore indicate that raised levels of pro-inflammatory cytokines may contribute to neurodegeneration in this group, or increased concentration of pro-inflammatory cytokines may be a protective response to neuronal damage. Raised IL1β concentration in adults with DS may also have implications for the development of microbleeds or strokes in response to cerebral amyloid angiopathy, as discussed in Buss et al. [59].

We did not find any associations with age and concentrations of Aβ peptides, tau, or cytokines in any group. Previous studies have reported contrasting results regarding the association between Aβ_42_ and age in DS, with some studies also finding no association [29, 32, 33, 60–62], some finding a positive association [34, 63, 64], and some finding a negative association [27, 30]. Previous studies have also suggested age shows a positive correlation with concentrations of Aβ_40_ [33, 34] and t-tau [38] in DS. It has further been suggested Aβ_42_ and Aβ_40_ concentrations may not show a linear relationship with age in DS, being stable until age 50 and then decreasing [65]. As reported previously, we found a positive association between age and NfL concentration in adults with DS [16]. Due to the strong association between age and the development of dementia in adults with DS, this may indicate NfL is a more suitable biomarker for dementia progression than Aβ or tau in this group. Further supporting the use of NfL as a potential biomarker for dementia progression in DS, our group and others have previously shown that NfL concentration is significantly higher in adults with DS and dementia compared to those with DS without dementia [16, 17].

Identifying a biomarker for dementia in DS is important for early detection and for monitoring disease progression and is required for clinical intervention studies in combination with cognitive tests sensitive to detecting cognitive decline [66]. In addition to NfL, several other potential plasma or urinary biomarkers have been proposed, including neopterin (a marker for activated cellular immunity and inflammation) [67, 68], 3-methoxy-4-hydroxyphenylglycol (MHPG, a noradrenergic metabolite) [69], dehydroepiandrosterone (DHEA, a steroid hormone) [70], or molecules associated with oxidative stress, specifically superoxide dismutase enzymes (SOD) [71] and iPF2alpha [72]. It has also been suggested that a combination of baseline concentrations and changes in Aβ peptides and inflammatory molecules may be predictive of cognitive decline in adults with DS [31]. Further longitudinal studies assessing multiple potential biomarkers with large samples and multiple time points starting prior to the onset of any cognitive decline are needed to clarify the ideal biomarker for dementia in DS. Blood samples are less invasive, easier, quicker, and cheaper to obtain than CSF samples, with additional practical considerations in those with DS often limiting the feasibility of obtaining CSF. The identification of a plasma biomarker for dementia progression in DS is therefore essential. Such a biomarker will be of particular use in the population with DS due to the pre-morbid variability in intellectual disability severity posing an additional challenge for the clinical diagnosis of dementia.

However, aside from plasma Aβ_42_ (and the Aβ_42_/Aβ_40_ ratio), where a reasonably robust association with cerebral β-amyloidosis has emerged [73], it is unknown how well plasma concentrations of molecules reflect changes within the brain, and the relative contribution of different sources (including the brain and platelets) to plasma concentrations is unknown. To better understand concentrations of Aβ_42_ and tau in the brain of adults with DS using blood samples, Hamlett et al. [74] measured concentrations from neuronal exosomes within the blood, finding higher concentrations of Aβ_42_ and phosphorylated tau in DS compared to controls. These results indicate an alternative to measuring plasma concentrations. Future studies are needed to further explore the relationships between plasma concentrations of molecules and changes within the brain.

Studies assessing blood biomarkers in DS typically consist of relatively small sample sizes [31, 38], in large part due to difficulties in obtaining blood samples from these individuals. Although our sample size is large enough to demonstrate group differences in plasma concentrations of some molecules, it is possible a larger sample would reveal additional group differences. It should also be noted we included individuals with varying stages of dementia progression in the group with DS, which may have contributed to the presence of some relationships in this group and not in the other groups, such as the positive associations between concentrations of t-tau and both Aβ_42_ and IL1β. Despite this, all adults with DS are thought to have Aβ neuropathology by their mid-30s [1, 7], and so the group with DS is likely similar in terms of the presence of Aβ neuropathology. Although some studies have suggested stage of dementia progression in DS may be associated with varying concentrations of Aβ_40_ and Aβ_42_ [28, 31, 32, 63, 75], other studies have reported no differences in Aβ_40_ or Aβ_42_ concentrations in those with DS with and without dementia [29, 31, 60–62, 76]. A recent study has further suggested that plasma Aβ_40_ and Aβ_42_ concentrations show poor diagnostic performance for dementia in DS [17]. Given these differences in results, which may be due to differences in study populations (including differences in age, dementia stage, or dementia duration) and assay sensitivity or sampling procedures, further larger, longitudinal studies with multiple time points starting prior to the onset of any cognitive decline and using ultrasensitive assays such as those used in the present study are needed to clarify whether changes in Aβ_40_ or Aβ_42_ concentrations are associated with the development and progression of dementia in DS.

Finally, considering the amyloid hypothesis of AD and associated changes in people with DS, our results support the triplication of *APP* leading to the overproduction of Aβ peptides and resulting in elevated concentrations of Aβ_40_ and Aβ_42_ in people with DS compared to the sAD and control groups. Within the group with DS, the positive relationship between NfL concentration and age (and also the previously published association between higher NfL concentration and dementia development [16, 17]) indicates plasma NfL concentration reflects neuronal damage in the brain. We did not find group differences in concentrations of t-tau or cytokines that may indicate altered concentrations with the development of dementia. It is possible our relatively small sample size limited our power to detect these differences. Alternatively, it is possible that the stage of dementia progression is relevant to detect group differences, and changes may occur in the earlier prodromal stage only. Again, larger, longitudinal studies with multiple time points starting prior to the onset of any cognitive decline would be required to determine associations between plasma molecular concentrations and the development of dementia.

## Conclusions

We compared plasma concentrations of Aβ peptides, tau, and selected cytokines between adults with DS, adults with sAD, and controls age-matched to the group with DS to investigate molecular mechanisms relevant to the development of AD in DS using ultrasensitive assays. Our results indicated the likely similar processing and deposition of Aβ_40_ and Aβ_42_ in those with DS and sAD, though those with DS showed much higher concentrations of these molecules, despite their younger age. In addition, IL1β concentration is far higher in those with DS compared to those with sAD and controls, and a positive association between IL1β and t-tau in those with DS may indicate IL1β is associated with neurodegeneration in this group. Our results also indicated that NfL concentration may be the most suitable biomarker for dementia progression in DS. Future longitudinal studies to identify biomarker changes over time associated with pathological and clinical progression are needed to confirm these findings.

## Abbreviations

AD: Alzheimer’s disease
APP: Amyloid precursor protein
Aβ: Amyloid-β
CSF: Cerebrospinal fluid
CV: Coefficient of variation
DHEA: Dehydroepiandrosterone
DS: Down syndrome
EMIF: European Medical Informatics Framework
IFN-γ: Interferon-γ
IL10: Interleukin 10
IL1β: Interleukin 1β
IL6: Interleukin 6
MHPG: 3-Methoxy-4-hydroxyphenylglycol
MMSE: Mini-Mental State Examination
N3PA: Neurology 3-Plex A assay
NfL: Neurofilament light
sAD: Sporadic Alzheimer’s disease
SOD: Superoxide dismutase enzymes
TNFα: Tumour necrosis factor α
t-tau: Total tau
